# Shouting strengthens maximal voluntary force and is associated with augmented pupillary dilation

**DOI:** 10.1038/s41598-021-97949-2

**Published:** 2021-09-16

**Authors:** Yudai Takarada, Daichi Nozaki

**Affiliations:** 1 Faculty of Sports Sciences, Waseda University, 2-579-15 Mikajima, Tokorozawa, Saitama 359-1192 Japan; 2 Graduate School of Education, The University of Tokyo, Tokyo, 113-0033 Japan

**Keywords:** Neuroscience, Motor control, Motor cortex

## Abstract

Previous research has demonstrated that human maximal voluntary force is generally limited by neural inhibition. Producing a shout during maximal exertion effort enhances the force levels of maximal voluntary contraction. However, the mechanisms underlying this enhancement effect on force production remain unclear. We investigated the influence of producing a shout on the pupil-linked neuromodulatory system state by examining pupil size. We also examined its effects on the motor system state by examining motor evoked potentials in response to transcranial magnetic stimulation applied over the contralateral primary motor cortex, and by evaluating handgrip maximal voluntary force. Analysis revealed that producing a shout significantly increased handgrip maximal voluntary force, followed by an increase in pupil size and a reduction of the cortical silent period. Our results indicate that producing a shout increased handgrip maximal voluntary force through the enhancement of motor cortical excitability, possibly via the enhancement of noradrenergic system activity. This study provides evidence that the muscular force-enhancing effect of shouting during maximal force exertion is related to both the motor system state and the pupil-linked neuromodulatory system state.

## Introduction

Maximal voluntary contraction (MVC) is the maximal force-generating capacity of a muscle or group of muscles in humans. In a pioneering study, Ikai and Steinhaus proposed that the MVC is limited by inhibiting mechanisms^1^. MVC has been found to be enhanced by various manipulations, including the sound of a gunshot^1^, hypnotic suggestion^1^, shouting^1,2^, and verbal encouragement^3^ during maximal exertion. These results indicate that maximum volition-induced motor system activity does not drive muscles to produce the full force of which they are capable, suggesting a latent ability for producing additional force that is hidden in ordinary force exertion. If this is the case, the MVC-enhancing manipulations described above are likely to enhance the excitability of the motor system. This hypothesis is supported by recent evidence that the subliminal priming of an action concept with a positive reward signal potentiates motor system activity, which enhances the maximal level of voluntary force exertion^4^.

The shouting protocol used to increase MVC in several previous studies^1,2^ is also a form of “psyching up,” a technique that can increase arousal to enhance physical performance via explosive force production^5^. Psyching up refers to self-directed cognitive strategies used immediately prior to or during skill execution, designed to enhance performance^6^. Many athletes who compete in tennis, power lifting, and other sports that require explosive movements undertake some form of psyching up during both training and competition^2,6^. However, the mechanisms underlying the enhancing effects of these methods on force production remain unknown. Listening to brief, loud sounds binaurally via headphones was reported to produce pupillary dilation^7^, suggesting that an increased level of arousal is achieved by enhancing sympathetic nerve system activity^8^. Importantly, few previous studies have explored the influence of shouting on neuronal activity in the brain (i.e., the state of the pupil-linked neuromodulatory system and/or the motor system) with special reference to human maximal force exertion.

Here, we investigated the influence of shouting on the pupil-linked neuromodulatory system state by examining pupil size^9,10^. We also examined the motor system state by examining motor evoked potentials (MEPs) in response to transcranial magnetic stimulation (TMS) applied over the contralateral primary motor cortex (M1), and by evaluating handgrip maximal voluntary force. Our results indicate that shouting can increase the handgrip force level of MVC through the reduction of motor cortical inhibition, accompanied by enhancement of pupil-linked neuromodulatory system activity. The current study provides evidence that the muscular force-enhancing effect of shouting during maximal force exertion is related to the enhancement of motor system activity, and the enhancement of pupil-linked neuromodulatory system activity.

## Methods

### Power analysis

We conducted an a priori power analysis to determine the required sample size for this experiment. We designed this experiment to have 80% power for detecting the effect sizes that we previously found for the influence of motivational goal-priming on the motor system and action (0.46–0.64, Cohen’s *d*)^4,9^ and/or pupil diameter (0.50–0.61, Cohen’s *d*)^9,10^, using a significance level of 5%. We used G*Power 3.1® (Institut für Experimentelle Psychologie, Düsseldorf, Germany) to compute the required sample size of the current study, which was 11 participants.

### Participants and procedures

Nineteen healthy Japanese right-handed individuals, as evaluated using the Edinburgh Handedness Inventory^11^, participated in the study. Participants included 18 men and 1 woman, with a mean age ± standard deviation of 20.4 ± 2.0 years. All participants provided both written and verbal informed consent. The study was conducted in accordance with the Declaration of Helsinki. All participants were university students with no clear description of strength training history, which suggests that they were untrained in exerting the maximal force generated briefly by a muscle or group of muscles at a specified speed. The experimental procedures complied with relevant laws and institutional guidelines, and were approved by the Human Research Ethics Committee of the Faculty of Sport Sciences of Waseda University (approval number: 2017-253).

**Figure 1:**
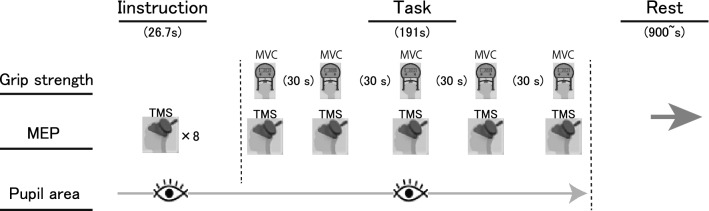
Experimental procedure. Each experiment consisted of two conditions (control and shout in MVC task phase), and each condition consisted of two phases (experimental instruction and MVC task). Each participant performed two conditions (control and shout) with a break of at least 15 min between each. The total experimental time was approximately 30 min. Instruction: experimental instruction phase. Task: MVC task phase.

Experiments were designed to examine the influence of a self-generated shout on handgrip maximal voluntary force, pupillary size, and MEP in the flexor carpi ulnaris (FCU) muscle in response to TMS (see *TMS* for details). Each experiment consisted of two conditions (control and shout), each with a duration of approximately 216 s, which included two phases: the experimental instruction phase, lasting for approximately 26 s, and the MVC task phase, lasting for approximately 190 s (Fig. 1). The total experimental period lasted approximately 30 min. Participants underwent two conditions (control and shout), with a break of at least 15 min according to the experimental instructions on a screen in front of them (see “Pupil diameter measurement” for details). The order of the two conditions was counterbalanced so that ten participants started with the control condition and the others started with the shout condition. In the shout condition, participants were asked to shout and squeeze a handgrip device with their maximal volition. Participants were not given detailed instructions regarding the loudness or duration of the shout. In contrast, in the control condition, participants were instructed not to shout. In both the control and shout conditions, participants were asked to keep their heads still and to keep their hands on their lap in a sitting posture while maintaining as much stability in the core as possible. All experimental procedures were conducted automatically via a 60-Hz cathode ray tube (CRT) screen that displayed the instructions in text form (see “Pupil diameter measurement” for details).

### Pupil diameter measurement

Pupil diameter was measured using a TalkEye Lite system (Takei Scientific Instruments Co., Ltd., Tokyo, Japan). An image around the pupil was obtained using a camera employing near-infrared light-emitting diodes and a video graphics array (640 × 480) (digital signal processor built in) camera module (NCM03-V, Nippon Chemi-Con Corporation, Tokyo, Japan). Banalization processing was performed on each image, and the pupil diameter was then measured according to the methods described by Wang et al.^12^. Changes in pupil size were estimated by the area of the pupil^9,10^ while participants viewed the experimental instructions (the experimental instruction phase) and exerted handgrip MVC (the MVC task phase) in the control and shout conditions. We calculated the average pupil area from the onset of the first word presentation to the disappearance of the last word in the experimental instruction phase, and during each number of squeezing a handgrip device displayed for 5 s (see “Handgrip force measurement”) in the MVC task phase.

The following steps were taken to exclude the impact of experimenter expectations for participant responses and measurements as much as possible, and to objectively estimate the effect of shouting. (1) All experimental procedures were conducted automatically using a 60-Hz CRT screen to display the text, and the experimental procedure was created using software designed for psychological experiments (Inquisit 3 Desktop Edition, Millisecond Software, Seattle, WA, USA). (2) All participants were instructed to follow the instructions on the screen only. (3) Pupil diameter measurements were automatically performed using a specially designed device with an eye-capturing camera to obtain the image around the pupil. Consequently, the paradigm used in the present study was less susceptible to experimenter bias compared with outcome measurements that have typically been used for examining MVC in previous studies^13^.

All word stimuli were displayed in black (20.8 cd/m^2^: mean value of five measurements of luminance with an LS160 luminance meter; Konica Minolta, Inc., Tokyo, Japan) on a white screen (124.2 cd/m^2^) during the experimental procedure. Immediately before the word presentation, the color of the screen was momentarily white without any black words. The pupil diameter may have transiently decreased because of the increase in luminance caused by the white screen with a maximum luminance of 129.6 cd/m^2^. Thus, we were unable to completely eliminate the possibility that this transient change in luminance affected pupil diameter. However, any effect would be likely to be minimal, because this phenomenon was common for all participants and conditions.

### Handgrip force measurement

**Figure 2:**
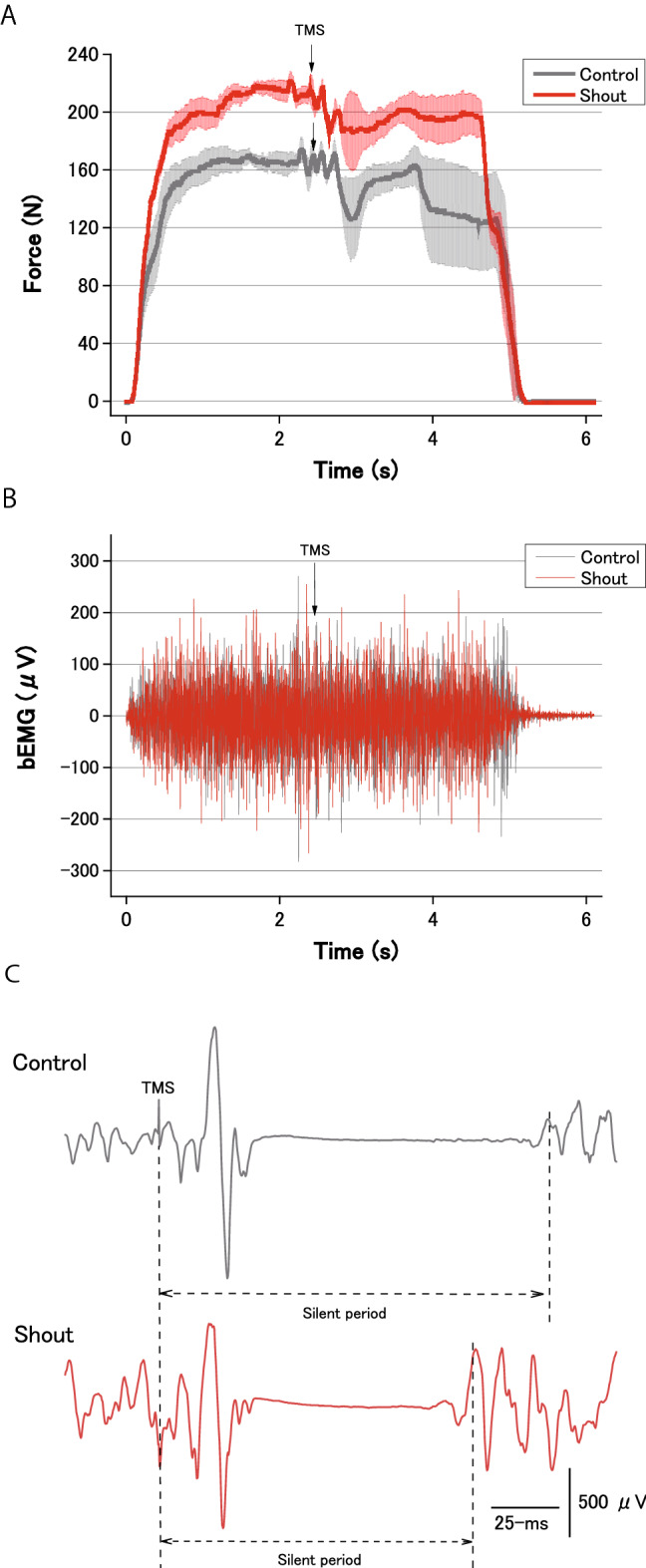
Typical recordings of handgrip force, background electromyography (bEMG), and typical motor evoked potential (MEP) waveforms of the flexor carpi ulnaris during the maximal voluntary contraction force (MVC) of handgrip in each experimental condition (control or shout) in a single participant. The timing of transcranial magnetic stimulation is indicated by the arrow. The handgrip force declined when transcranial magnetic stimulation was delivered during the contraction, the timing of which was different in each contraction. **(A)** Data of force are expressed as the mean ± standard error of the mean of five recordings. **(B)** bEMG during handgrip contraction for each condition. **(C)** Duration of the cortical silent period during handgrip contraction for each condition.

Force was measured using a handgrip device (KFG-5-120-C1-16, Kyowa Electronic Instruments, Tokyo, Japan). The experimental instructions on the screen asked participants to squeeze the handgrip device with the right (dominant) hand with their maximum effort when each number indicating “1st time” to “5th time” appeared, and to stop squeezing when the number disappeared. The handgrip device was fixed to the right thigh with an elastic band so that the device did not move when it was squeezed by the participant. The number was displayed for 5 s. This was repeated five times, with a 30-s inter-squeeze interval. The maximal values of the exerted force were averaged from the 500-ms steady state of the force curve before each TMS to the 100 ms state of the force curve after each TMS according to the methods described by Gandevia et al.^14^ across the five trials. This was defined as the handgrip MVC (Fig. 2A).

### TMS

In both the control and shout conditions, single-pulse TMS was administered via a stimulator (M2002, Magstim, Whitland, UK) using a double figure-eight-shaped coil (4150-00 Double 70 mm Alpha Coil, Magstim) with a maximum magnetic field strength of 1.55 T. Each participant sat upright with their elbows bent in front of them, resting on their thighs. The TMS coil was then positioned over the finger area of the left M1, which was determined as the area with the lowest resting motor threshold (rMT). This was defined as the area for which MEPs with peak-to-peak amplitudes greater than 50 µV were induced in the FCU muscle^9,15,16^ in at least five out of ten trials when participants were fully relaxed with their eyes closed^17^. The coil position was stabilized throughout the experiment using a coil stand made from multiple products (Manfrotto Distribution KK, Tokyo, Japan). The optimal scalp position of M1 was marked directly onto the scalp with a black marker pen. The positioned coil was monitored continuously to maintain consistent positioning throughout the experiment. The rMTs ranged from 50 to 70% of the maximum stimulator output, and the stimulus intensity for each participant was set at 110% of their rMT while viewing the experimental instructions. The stimulus intensity was set from 70 to 90% of the maximum stimulator output during handgrip force exertion. The stimulation was automatically delivered eight times at 3-s intervals during the experimental instruction phase. Thus, MEPs were recorded eight times for each condition (control or shout). Stimulations were manually delivered over the target site during each 5-s MVC, with a 30-s inter-squeeze interval (Fig. 2A), the timing of TMS was different for each 5-s MVC in the MVC task phase. Thus, the MEP was recorded five times for each condition (control or shout). The TMS intensity was fixed for each participant. Surface electromyograms were obtained from the right FCU muscles via bipolar silver surface electrodes (bandpass, 15 Hz–10 kHz) using the tendon-belly method^4,9^.

### Background EMG and MEP measurement and analysis

When the background EMG (bEMG) activity was high (Fig. 2B), it was difficult to discriminate the MEP in a single trace. We therefore calculated the averaged waveform of MEP (an average of eight recordings in the experimental instruction phase, and an average of five recordings in the MVC task phase for each condition evoked by TMS) to reduce the bEMG^16,18^. For each condition, we thus calculated the peak-to-peak amplitude of the averaged MEP across eight recordings in the experimental instruction phase, and across five recordings in the MVC task phase. To measure the bEMG, a rectified EMG signal of the period approximately 100 ms before TMS was integrated, during which the force was kept at the maximum force level (Fig. 2A,B). The duration of the cortical silent period was taken as the time interval from the stimulus artifact to the return of continuous EMG^19,20^ (Fig. 2C). When it was difficult to determine the end of the cortical silent period (because voluntary EMG activity does not recover abruptly, but rather recovers gradually), the end of the cortical silent period was determined when the corresponding rectified EMG activity reached a value within two standard deviations of the rectified EMG signal of the period approximately 100 ms before TMS^21,22^.

### Statistical analysis

Statistically significant differences in handgrip MVC, the duration of the cortical silent period, and bEMG between the control and shout conditions were investigated using paired *t*-tests. MEP amplitude and pupil area were analyzed using repeated-measures two-way analyses of variance with within-participant factors of Condition (control or shout), and Phase (experimental instruction or MVC task). Greenhouse–Geisser corrections were applied when appropriate to adjust for non-sphericity, changing the degrees of freedom using a correction coefficient. Post hoc analysis used paired *t*-tests for each experimental condition (control or shout). A significance threshold of *P* < 0.05 was chosen for all tests.

## Results

### Handgrip force

**Figure 3:**
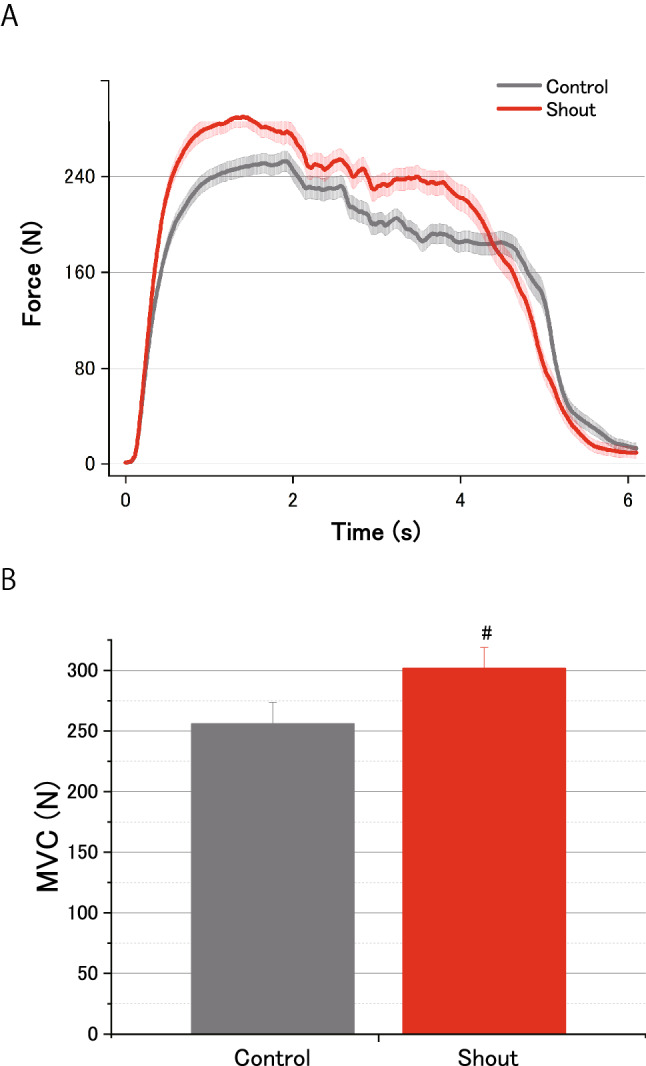
Effects of shouting on the maximal voluntary contraction force (MVC) of handgrip. **(A)** Typical recordings of handgrip force in each experimental condition (control or shout), which were averaged across all participants. **(B)** The averaged MVC across the five handgrip trials for the two conditions. The shout condition resulted in a greater handgrip MVC compared with the control condition. Data are expressed as the mean ± standard error of the mean (^#^*P* < 0.01, paired *t*-test).

First, we replicated the previous finding that shouting was associated with enhanced MVC (Fig. 3A). The handgrip MVC was significantly greater in the shout condition (304.4 ± 16.6 N) compared with the control condition (259.7 ± 16.9 N) (Fig. 3B; *t*(18) =  − 6.22, *d* = 0.61; *P* = 7.18 × 10^–6^).

### MEP

**Figure 4:**
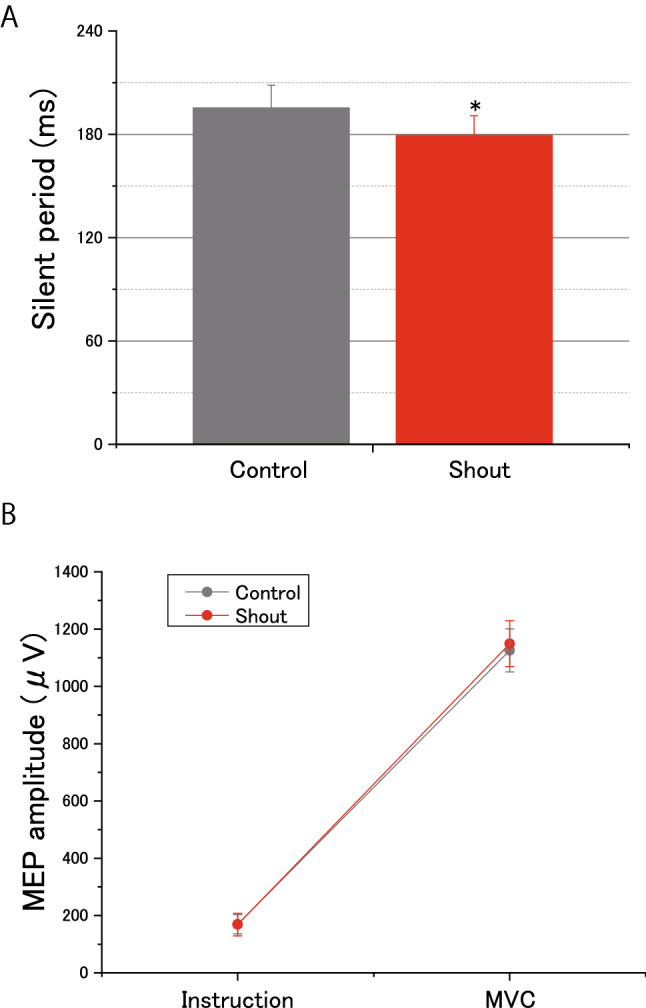
Effects of shouting on the cortical silent period and motor evoked potential (MEP) amplitude. **(A)** Durations of the cortical silent period for the two conditions during the maximal voluntary contraction force (MVC) of handgrip. The duration of the cortical silent period in the shout condition was significantly shorter than that in the control condition (*P < 0.05, paired *t*-test). **(B)** Amplitudes of MEPs of the flexor carpi ulnaris during the experimental instruction and MVC task phases for the two experimental conditions (control or shout). There was no significant difference in MEP amplitude between the two conditions. Data are expressed as the mean ± standard error of the mean.

Compared with the control condition (195.3 ± 13.1 ms), the duration of the cortical silent period during handgrip MVC was reduced in the shout condition (179.3 ± 11.4 ms). A paired *t*-test revealed that this reducing effect of shouting was indeed significant (Fig. 4A, t(18) = 2.68, *d* = 0.30; *P* = 0.0015). However, there were no significant differences in MEP amplitudes between the two conditions during the experimental instruction (control: 170.0 ± 33.7 µV; shout: 168.6 ± 39.7 µV) or MVC task (control: 1125.8 ± 75.1 µV; shout: 1149.3 ± 79.8 µV) phases. A two-way analysis of variance revealed a significant main effect of Phase (*F*(1,18) = 195.10; *P* = 4.22 × 10^–11^; effect size: partial *η*^2^ = 0.91), but no significant effect of Condition (*F*(1,18) = 0.18; *P* = 0.67; effect size: partial *η*^2^ = 0.01), and no interaction between Condition and Phase (*F*(1,18) = 0.17; *P* = 0.68; effect size: partial *η*^2^ = 0.01; Fig. 4B). Background electromyography (EMG) revealed no significant changes among the conditions during the experimental instruction (*t*(18) =  − 0.62, *d* = 0.12; *P* = 0.53) and MVC task (*t*(18) =  − 1.03, *d* = 0.10; *P* = 0.31) phases.

### Pupil area

**Figure 5:**
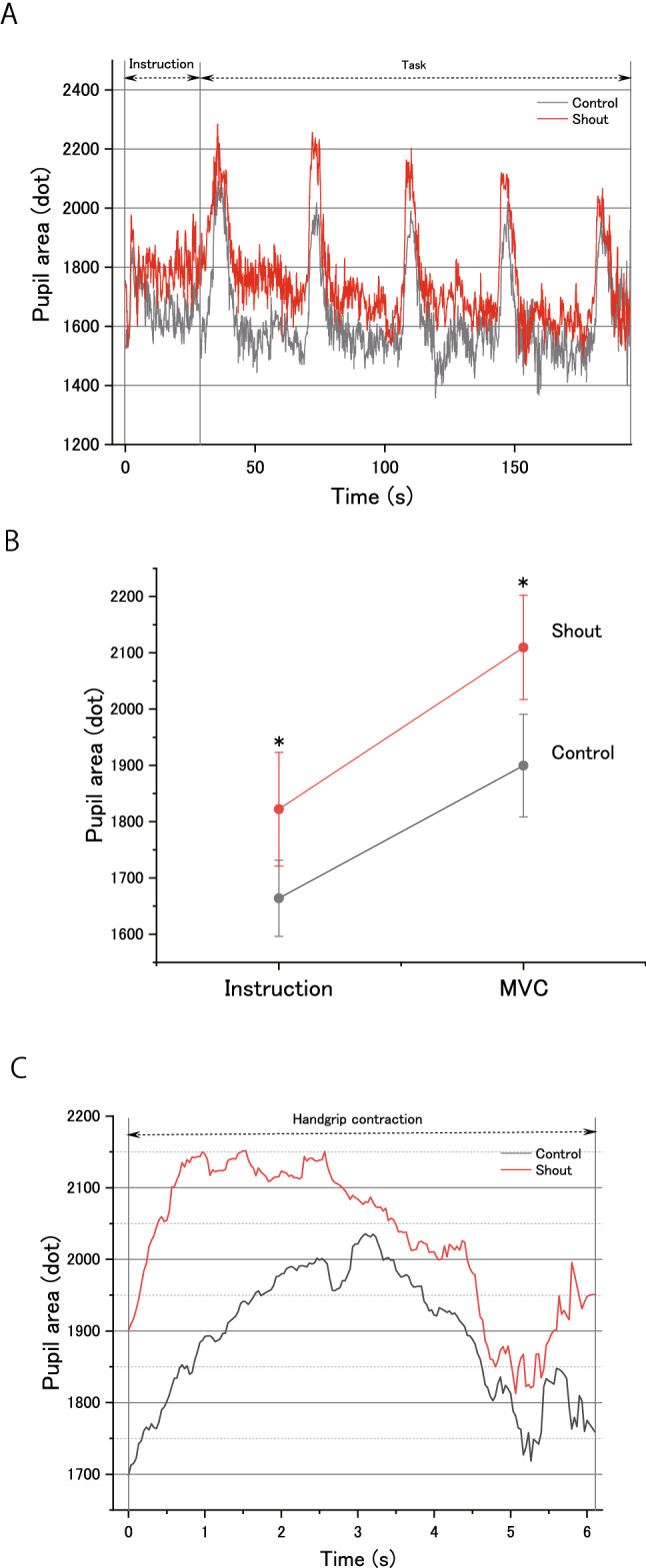
Effects of shouting on pupil area over time. **(A)** Pupil area (dots) starting at the onset of word presentation in the experimental procedure and lasting until the end of the maximal voluntary contraction force (MVC) task. Pupil area is expressed as the mean for each experimental condition (control or shout). The data were low-pass filtered with a cut-off frequency of 1 Hz using a fourth-order Butterworth filter. Two bidirectional arrows ( ↔) indicate the period of the experimental instruction and the period of the MVC handgrip task, respectively. Instruction: experimental instruction phase. Task: MVC task phase. Pupil area (dots) is expressed as the mean during the period of the experimental instruction phase and during MVC (not during the MVC task phase) for each experimental condition (control or shout). **(B)** Pupil area (dots) during the period of the experimental instruction phase and during each number of squeezing a handgrip device displayed for 5 s (not during the MVC task phase) for the two experimental conditions (control or shout). (C) Averaged pupil area (dots) during squeezing a handgrip device displayed for 5 s for the two experimental conditions (control or shout). The shout condition resulted in a greater pupil area compared with the control condition. However, the result does not necessarily guarantee that the pupillary dilation by shouting causally related to the MVC enhancement: it could reflect that the pupil diameter just responded to shouting and MVC. Pupil area data **(A,B)** are expressed as the mean ± standard error of the mean. *P < 0.05, vs. control condition.

Figure 5A shows the time course of pupil area measurements from the onset of the experimental instruction phase to the end of the MVC task phase. Pupil size increased during the period of the experimental instruction phase and during squeezing the handgrip device, displayed for 5 s in the MVC task phase for the two experimental (control and shout) conditions. Analyses revealed significant main effects of Condition (*F*(1,18) = 30.12; *P* = 3.26 × 10^–5^; effect size: partial *η*^2^ = 0.62) and Phase (*F*(1,18) = 26.8; *P* = 6.24 × 10^–5^; effect size: partial *η*^2^ = 0.59), but no significant interaction between Condition and Phase (*F*(1,18) = 0.74; *P* = 0.39; effect size: partial *η*^2^ = 0.04). Post hoc analyses revealed a significantly larger change in pupil area in the shout condition compared with the control condition during the experimental instruction phase (*t*(18) =  − 3.11, *d* = 0.42; *P* = 0.006) (Fig. 5B) and while squeezing the handgrip device in the MVC task (*t*(18) =  − 5.03, *d* = 0.52; *P* = 8.60 × 10^–5^) phase (Fig. 5C).

## Discussion

In the present study, the results demonstrated that shouting significantly increased the handgrip force level of MVC, followed by an increase in pupil size and a reduction of the cortical silent period. Such an enhancing effect of shouting on handgrip MVC is generally consistent with results of previous studies^1,2^. Our findings indicate that the pupil-linked neuromodulatory system and the motor system were more excitable during muscular contraction paired with shouting, resulting in the production of additional muscular force in maximal exertion effort. These results indicate that maximum volition-induced motor system activity does not drive muscles to produce the full force of which they are capable, and that there is a latent ability for producing additional force that is hidden in ordinary force exertion.

### Enhancement effect of shouting on handgrip MVC

The main finding of the current study was the marked enhancement effect of shouting on handgrip MVC, with an average increase in the rate of handgrip MVC of approximately 15%. This rate of increase is greater than the effect of shouting on forearm flexion MVC (12%) previously reported in a study by Ikai and Steinhaus^1^, and the effect on MVC of kiap (9.5%), a psyching-up technique similar to shouting that is used in martial arts, reported in a study by Welch and Tschampl^2^. One reason for this discrepancy is that our participants were university students with no clear training history, whereas the participants in Welch and Tschampl’s study^2^ had martial arts experience and regularly undertook martial arts training. Trained individuals may not be readily modifiable by a psyching-up technique like kiap because of their well-developed movement patterns and neural pathways, whereas untrained individuals may be more readily modifiable by shouting because of their less-developed movement patterns and neural pathways^23^. Even conventional resistance exercise training with high-intensity mechanical stimuli is unlikely to readily cause an increase in MVC with the same magnitude of increase in maximal neural activation as that in MVC in trained athletes^24^. Unfortunately, because Ikai and Steinhaus^1^ did not report the detailed training history of their participants, it is not possible to compare and assess the magnitude of the enhancing effect of shouting on MVC between their study and our current research. Thus, the differences in the enhancing effects on MVC mentioned above may be related to the familiarity with the psych-up strategy and the training history of each individual.

### Pupil size as an index of intensity of handgrip contraction

Pupillometry has long been used as a measure of brain state. A number of studies have reported that pupillary dilation is related to mental effort (cognitive load), and the correspondence between cognitive load and pupillary dilation has been documented in several contexts, including paired-associate learning^25^. Pupil size increases according to the complexity of the mental task^25^. A recent study has demonstrated that the pupil size also increases during physical effort, the degree of which reflects the actual intensity of muscular contraction^26^. Thus, we examined pupil size during handgrip MVC between the shout and control conditions because we consider that the effects of noradrenaline (NE) on the activity of motor cortical neurons (as described below) can be indirectly estimated by pupil size. This notion is supported by several previous studies^27–29^.

We were unable to completely eliminate the possibility that transient changes in the luminance of the screen and/or the appearance of the number of handgrip squeezes affected the pupil diameter during handgrip MVC. However, the effect of this type of contamination would be expected to be small because such transient changes in the luminance was common across all participants, and the magnitude of increase in pupil size during the instruction phase including the appearance of the number was smaller than that during the handgrip MVC (see “Relationship between shouting and pupillary dilation” for details).

### Enhancement effects of shouting on motor system activity through the potentiation of the pupil-linked neuromodulatory system

In the current study, shouting significantly increased pupil size and reduced the cortical silent period. Changes in pupil diameter are thought to correspond to the activity of neuromodulators, including NE and acetylcholine, which produce alterations in the brain state and corresponding changes in behavior. It is currently unknown whether activity in only noradrenergic locus coeruleus (LC) neurons directly influences pupil size; however, noradrenergic neurons are reported to be active during pupillary dilation^30^. Changes in silent periods of longer than 100 ms, as recorded in the hand muscles of healthy participants in one previous study^31^, are considered an index of cortical inhibition^19^. The site of origin of the cortical silent period is largely in the M1^19^, in which GABABergic circuits are thought to generate the cortical silent period^32–34^. Thus, a reduced duration of the cortical silent period, as observed in the current study (Fig. 4A) might be associated with the enhancing effects of NE on the activity of motor cortical neurons^35–40^.

Despite the reduction of the cortical silent period, we failed to detect any changes in MEP amplitude during MVC; there were no significant differences in MEP amplitudes between the shout and control conditions (Fig. 4B). This failure to detect any changes might be associated with recruitment of the M1 neurons to reach a plateau level during MVC. In other words, most of the M1 neurons may have already been recruited^41^, leaving fewer neurons available to respond to TMS. Thus, the level of M1 neuron recruitment reaching a plateau during MVC might have overshadowed any differences in MEP amplitudes between the shouting and control conditions.

### Relationship between shouting and pupillary dilation

The production of shouting necessitates two pathways, which are organized hierarchically, building from the basic levels in the lower brain stem and spinal cord to the most complex levels in the anterior cingulate cortex (ACC) and the laryngeal motor cortex (LMC), respectively^42^. In the present study, participants in the shout condition were asked to shout and perform handgrip MVC (see “Participants and procedures”). Coordination and interactions between the LMC and ACC-periaqueductal gray (PAG) pathways are indispensable for proper voice control and voice initiation in the shout condition (see ref.^42^ for details). Taking into account reciprocal connections of the LMC not only to motor cortices, but also subcortical regions including LC^42,43^, shouting may stimulate the activity of LC neurons, possibly by enhancing the activity of the LMC and ACC–PAG pathways, which results in pupillary dilation. Moreover, as mentioned in the Introduction, shouting-induced noise (loud sound) may have influenced pupil dilation in our study^7^. Because sound stimuli have an arousal effect, which is benefited in unplanned actions more than in planned ones^7^. However, we consider that such an effect of noise on pupil dilation would not be able to conceal the increase in pupil area in the shouting condition because participants were never given instructions regarding loudness in the shout condition. In the experiment, the loudness of the noise when shouting differed among participants in the shout condition.

Pupillary dilation was not observed during the MVC task phase, but also was observed during the experimental instruction phase (Fig. 5). We speculate that the cause of pupillary dilation during the experimental instruction phase was as follows. First, pupillary dilation during this phase may have been caused by motor imagery when performing the handgrip MVC combined with a self-generated shout immediately after the instruction was given. When debriefed, participants reported that they imagined the combined motor action. Some previous studies reported motor imagery-induced pupillary dilation^44–46^. Although each motor action (i.e., handgrip or shout) is relatively simple, the combined motor action execution requires the processing of higher-order motor control (see the previous descriptions of central shouting control). Thus, motor imagery during the instruction phase may induce pupillary dilation. Another cause may be related to the time pressure associated with the motor imagery: participants had to complete the rehearsal activity before the text “1st time” was unpredictably presented on the monitor, prompting participants to start squeezing the handgrip device immediately after seeing the experimental instruction. Some previous studies reported that such time pressure is inherent in the structure of a mental task and induces particularly large pupillary dilations^44,47^. Thus, time pressure associated with motor imagery may have induced pupillary dilation in the current study.

Consequently, we cannot exclude the possibility that pupil-dilating effects during the experimental instruction phase might have influenced pupillary dilation during the handgrip MVC phase with shouting. However, the percentage increase in pupillary dilation during MVC (12.1 ± 2.7%) was much greater than that during experimental instruction (8.5 ± 2.9%) (Fig. 5). Thus, a change in pupillary dilation during the experimental instruction phase cannot adequately explain the difference in pupillary dilation across tasks. We therefore consider that the pupillary dilations in the experimental instruction and MVC task phases had different causes.

## Conclusion

In the current study, shouting led to a reduced cortical silent period with dilated pupils during MVC, and increased handgrip maximal voluntary force levels. Increased MVC may have been caused by the reduction of motor cortical inhibition, possibly via potentiation of the pupil-linked neuromodulatory system. These results indicate that maximum volition-induced motor system activity did not drive muscles to produce the full force of which they are capable. In turn, this suggests that a fluctuating factor of MVC is an active characteristic of the neural systems in the human brain, and that maximum volition does not cause the motor system to produce maximum activity. It should be noted, however, that the current finding does not necessarily guarantee that the pupillary dilation by shouting causally related to the MVC enhancement: it could reflect that the pupil diameter just responded to shouting and MVC. It is necessary for future studies to examine the causal links between the pupil-linked neuromodulatory system and maximal force development, with special reference to the motor system.
